# Metabolic Abnormalities, Dietary Risk Factors and Nutritional Management in Amyotrophic Lateral Sclerosis

**DOI:** 10.3390/nu13072273

**Published:** 2021-06-30

**Authors:** Emanuele D’Amico, Giuseppe Grosso, Jeri W. Nieves, Aurora Zanghì, Pam Factor-Litvak, Hiroshi Mitsumoto

**Affiliations:** 1 Department G.F. Ingrassia, University of Catania, 95123 Catania, Italy; emanuele.damico@unict.it (E.D.); aurora.zanghi@yahoo.it (A.Z.); 2 Department of Biomedical and Biotechnological Sciences, University of Catania, 95123 Catania, Italy; 3 Mailman School of Public Health and Institute of Human Nutrition, Columbia University, New York, NY 10032, USA; jwn5@cumc.columbia.edu (J.W.N.); prf1@cumc.columbia.edu (P.F.-L.); 4 Eleanor and Lou Gehrig ALS Center, The Neurological Institute of New York Columbia University Medical Center, New York, NY 10032, USA; hm264@cumc.columbia.edu

**Keywords:** amyotrophic lateral sclerosis, neurons, glucose metabolism, lipid metabolism, obesity, metabolic disorders, energy expenditure, dietary factors, malnutrition, comprehensive review

## Abstract

Amyotrophic Lateral Sclerosis (ALS) is a devastating progressive neurodegenerative disease that affects motor neurons, leading to a relentless paralysis of skeletal muscles and eventual respiratory failure. Although a small percentage of patients may have a longer survival time (up to 10 years), in most cases, the median survival time is from 20 to 48 months. The pathogenesis and risk factors for ALS are still unclear: among the various aspects taken into consideration, metabolic abnormalities and nutritional factors have been the focus of recent interests. Although there are no consistent findings regarding prior type-2 diabetes, hypercholesterolemia and ALS incidence, abnormalities in lipid and glucose metabolism may be linked to disease progression, leading to a relatively longer survival (probably as a result of counteract malnutrition and cachexia in the advanced stages of the disease). Among potential dietary risk factors, a higher risk of ALS has been associated with an increased intake of glutamate, while the consumption of antioxidant and anti-inflammatory compounds, such as vitamin E, n-3 polyunsaturated fatty acids, and carotenoids, has been related to lower incidence. Poor nutritional status and weight loss in ALS resulting from poor oral intake, progressive muscle atrophy, and the potential hypermetabolic state have been associated with rapid disease progression. It seems important to routinely perform a nutritional assessment of ALS patients at the earliest referral: weight maintenance (if adequate) or gain (if underweight) is suggested from the scientific literature; evidence of improved diet quality (in terms of nutrients and limits for pro-inflammatory dietary factors) and glucose and lipid control is yet to be confirmed, but it is advised. Further research is warranted to better understand the role of nutrition and the underlying metabolic abnormalities in ALS, and their contribution to the pathogenic mechanisms leading to ALS initiation and progression.

## 1. Introduction

Amyotrophic lateral sclerosis (ALS) is a neurodegenerative disease characterized by the degeneration of lower and upper motor neurons, which leads to progressive skeletal muscle atrophy, paralysis, and consequent death, usually with a 48-month medium survival after symptom onset [1]. The ALS affects approximately two individuals per 100,000 each year, with a prevalence of about seven individuals per 100,000. Males are slightly more affected than females (ratio of about 1.6:1) [2]. As clinical phenotypes are markedly heterogeneous, ALS started to be considered as a “syndrome” rather than a single nosological condition [3]. While most ALS cases (more than 90%) are sporadic, from approximately 5 to 10 percent of all ALS are related to multigenic traits [4]; however, the overall genetic background of the disease is only partly understood.

Putative disease mechanisms include several cellular changes, starting with neuroinflammation and oxidative damage, leading to mitochondrial dysfunction, the accumulation of intracellular protein aggregates, impairment of axonal transport, growth factor abnormalities, glutamate excitotoxicity, etc. [5]. Among the potential risk factors, a host of environmental factors were considered [6]. Moreover, emerging evidence suggests that the microbiome may be linked to neurodegenerative diseases, and directly related to nutrition and energy metabolism [7], and, interestingly, an alteration in the microbiome profile was shown in ALS patients [8]. Although the pathological changes characterizing the abnormalities in ALS were considered to only affect motor neurons, it appears that the wider involvement of the central nervous system (CNS), such as the frontotemporal lobar degeneration (FTLD) and pseudobulbar affect, as well as the implication of other districts, such as the skin, bone, gastrointestinal, and immune system, may occur [9]. This broader involvement could explain the vast diversity of the neuropathological and clinical phenotypes associated with ALS, raising the necessity of considering all the systemic effects of the disease when formulating the therapeutic interventions [10,11].

Since its approval in December 1995, Riluzole, an anti-glutamate agent able to extend the survival of ALS patients by a few months, has remained the only treatment [12]. In 2017, the second drug approved by the US Food and Drug Administration (FDA) edaravone was an antioxidant free-radical scavenger; however, it has not been approved by European Medicine Agency (EMA). A study on Japanese patients receiving it revealed that they declined less rapidly than those receiving a placebo, as measured by the change in the revised ALS Functional Rating Scale (ALSFRS-R) score after 24 weeks of treatment following an untreated 12-week lead-in [13]. However, another three trials on edaravone reported adverse outcomes [14]. Furthermore, it has antithrombotic and thrombolytic properties, and its relative benefit might reflect the mitigation of infusion-related thrombosis [15]. Although it is possible to better manage patients with ALS than before [16,17,18], disease-modifying medications have been severely limited.

Evidence suggests that ALS patients may suffer from an abnormal energy metabolism, as well as an impaired glucose and lipid metabolism [19,20,21]. Furthermore, body weight and body mass index (BMI) have been hypothesized to play a role in disease occurrence and progression in ALS [22]. Diet has been shown to potentially play a role in less disruptive mental health disorders [23], but there is a growing interest in exploring the potential role of nutritional and metabolic factors in affecting the survival of ALS patients [24]. The aim of this study is to review the current evidence on the role of metabolism and nutrition in the etiology and prognosis of ALS on human subjects.

## 2. Metabolic Changes in ALS

Metabolic abnormalities, including dyslipidemia and glucose intolerance, have often been reported in patients with ongoing ALS [25] and pre-morbid conditions [26]. However, it is unclear whether the metabolic changes found in ALS patients only arise as a result of neurodegeneration or whether they might play a role in the pathogenesis of the disease [21].

### 2.1. Glucose Metabolism in ALS

Earlier studies reported no difference [27,28] or higher basal insulin levels in ALS compared to control patients [29]. In the last decade, new evidence relating type-2 diabetes mellitus (T2DM) and ALS has been reported [30]. In a large clinical cohort (*n* = 2371), T2DM was associated with lower risk of developing ALS [31,32]; moreover, in those patients where ALS developed, a delay of 4 years in the onset of the disease was observed [33]. Since then, several studies have confirmed a potential protective role of T2DM in ALS in terms of delayed onset [34,35] and increased survival with T2DM [36], although the findings are not entirely consistent [37,38]. Another study investigating pre-morbid glucose abnormalities in ALS patients showed that the occurrence of insulin-dependent type-1 diabetes was associated with a higher risk of ALS in younger individuals, while T2DM was inversely associated with risk of ALS in older ones [39]; it has been hypothesized that genetic differences and lifelong medications may play a role, although the exact mechanisms are unclear [40].

There is evidence of impairment in the glucose-metabolizing pathways in ALS [41], such as decreased glucose consumption in the frontal lobe and cortex of patients with ALS [42,43]. Deficits in the insulin-mitochondrial axis could help explain the glucose metabolism abnormalities that have been described in ALS patients (see Table 1). The abnormal glucose intolerance in ALS could be secondary to muscle wasting or physical inactivity in ALS [44]. Another possibility is that there may be a decreased ability to store and/or mobilize loaded glucose in muscle [45]. Hyperlipidemia, also described in ALS, may also modulate glucose metabolism [46].

The metabolism of the energetic substrates (glucose and lipids) is closely related to the distribution of fat in the human body, and increased visceral fat is associated with T2DM, dyslipidemia and insulin resistance [60]. Earlier studies on adipose tissue in ALS patients focused on an analysis of fat mass (FM) using bioelectrical impedance analysis, showing that a decrease in fat-free mass was associated with adverse outcomes (including shorter survival) regardless of weight loss [61,62,63]. In a study using Magnetic Resonance Imaging (MRI)-based methodologies involving 62 ALS patients and matched controls, the distribution of total FM appeared to be roughly similar between groups, but ALS patients had expanded visceral fat and a higher visceral to subcutaneous fat ratio compared to the control [64].

Energy balance involves many other components, such as endocrine hormones (e.g., thyroid hormones, insulin/glucagon system, growth factor), but none of these were found to be abnormal in ALS [65,66]. Overall, glucose metabolism seems to be altered in ALS patients, but these abnormalities are not constantly observed in all patients [67,68,69]. Thus, these abnormalities cannot be consistently used as independent predictors of ALS prognosis.

### 2.2. Lipid Metabolism

The CNS is characterized by the variety and amount of lipids necessary to control membrane fluidity and the transmission of electrical impulses, and serve as precursors for second messengers [70]. Moreover, they are considered crucial to maintain the motor neuron functionality, with alterations in their homeostasis leading to potential neurodegenerative diseases [71].

The role of lipids in ALS prognosis is still unclear and the findings are rather conflicting [72]. Older studies showed alterations in circulating blood cholesterol (and related lipoprotein particles) and triglycerides over the course of ALS [47]. In a study conducted on 369 patients with ALS compared to a matched healthy control group, the frequency of hyperlipidemia (increased plasma levels of total and low-density lipoprotein (LDL) cholesterol), was more frequently observed in ALS patients than in control subjects [48]. In contrast, another study showed that the serum cholesterol and LDL/HDL ratio were significantly lower in controls than in ASL patients [55], and that baseline levels of cholesterol and LDL were inversely correlated with declined functionality in ALS patients [53]. Lipid alterations were also investigated in patients with ALS with or without cognitive and behavioral change (with the criteria of Frontotemporal Dementia (FTD)) [73]. In a case-control study of 128 participants, including 96 ALS patients and 32 healthy controls, an increased triglycerides (*p* < 0.001), total cholesterol/HDL ratio (*p* < 0.001), and lower HDL levels (*p* = 0.001) were observed in the cases group compared to the controls; moreover, a higher cholesterol level was associated with 3-fold longer survival (*p* = 0.008) [74]. The association between pre-diagnostic lipid levels and ALS risk was studied in a large nested case-control study involving 275 individuals from five prospective US cohorts (the Nurses’ Health Study, the Health Professionals Follow-up Study, the Cancer Prevention Study II Nutrition Cohort, the Multiethnic Cohort Study, and the Women’s Health Initiative): after a follow-up period, higher ALS risk was associated with higher HDL, while null results were reported for the other plasma lipids (including TC, LDL-C, and TG) [75].

Concerning survival, patients with ALS who had high LDL/HDL ratios lived longer than those with a low LDL/HDL ratio [48]. Several other studies reported that ALS patients with dyslipidemia or higher cholesterol levels were more likely to have a longer survival rate than those with no lipid abnormalities [48,50,56,57,58,59,76,77]. However, others found no association between plasma lipid levels and ALS survival [51,54,78], although a decrease in LDL/HDL ratio has been reported to be associated with a high rate of respiratory impairment [49].

A possible common pathologic pathway linking dyslipidemia to ALS could involve the mitochondrial system, which could explain the impaired energy balance seen in ALS. In ALS, there is accumulating evidence that mitochondrial dysfunction may play a significant role in the disease mechanisms [79,80,81]. In an older study, intracellular analysis of the hepatocytes studied in 21 liver biopsies, obtained from ALS patients, revealed a number of alterations, including an abnormal mitochondria with paracrystalline inclusions, abnormal lamellar structure of rough endoplasmic reticulum, higher number of smooth endoplasmic reticulum, and para-sinusoidal fibrosis [82]. Moreover, postmortem histologic examinations have also shown a more pronounced steatosis of liver in ALS patients (despite weight loss) than in other neurological diseases [48]. Mitochondrial dysfunction has been associated with insulin resistance in skeletal muscle and insulin-resistant individuals show characteristic changes from the normal lipid and lipoprotein pattern [83]. These changes include the elevation of triglycerides, reduction in HDL, and a concomitant increase in LDL [75]. The presence of dyslipidemia in ALS could reflect an intrinsic abnormality or a physiological attempt to compensate for an abnormal metabolic state [84]. The understanding of such evidence and the mechanisms underlying these abnormalities are not clear, but it may be important to monitor blood lipids to assess the risk of survival [72].

## 3. BMI Changes in ALS

BMI is a surrogate measure to identify an excess in weight, adjusted for height, and calculated as weight in kilograms divided by the square of height in meters (kg/m^2^) [85]. A recent pooled analysis conducted on about 400 thousand women (428 ALS deaths) and nearly 150 thousand men (204 ALS deaths) from 10 community-based cohorts in the USA, Europe and Australia, aiming to explore the relationships between ALS death and preclinical anthropometric measures, showed that weight-gain during adulthood was a strong predictor for lower mortality risk from ALS [86]. In a case-control study conducted on 279 individuals, subjects with motor neuron diseases (*n* = 279) were more likely to report they had always been slim compared to controls [87]. A retrospective study assessing BMI and cholesterol levels in 427 ALS patients found that a decrease in BMI was associated with lower survival, irrespective of other comorbidities (including cardiovascular disease) associated with a high BMI [52].

A more recent prospective cohort study (enrolling 537,968 females and 562,942 males) recorded weight at time of enrollment, recalled weight at age 18/21 years, and were followed over 14–28 years; among the 1153 participants who developed ALS during the follow-up period, ALS rates were significantly lower among the overweight (odds ratio (OR) 0.76; 95% CI, 0.62 to 0.93) and obese (OR 0.73; 95% CI, 0.55 to 0.96) individuals compared to those with normal BMI, while being underweight was associated with higher risk of ALS (risk of ALS for a 5-unit increase in BMI was 0.79 (95% CI: 0.73 to 0.86, *p <* 0.0001)) [88].

In another prospective study [89], 222 ALS deaths occurred in the European Prospective Investigation into Cancer and Nutrition cohort study (involving 518,108 individuals) after a 13-year follow-up period, registering a higher mortality risk from ALS in underweight individuals (HR = 2.79, 95% CI 1.35 to 5.77), while no association was found for the analyses of waist and hip circumferences and waist/hip ratio with ALS mortality [89].

A BMI decrease in ALS may depend on both a loss in FM, but also in muscle atrophy [90]. Among the factors that could contribute to changes in BMI, there are changes in sensory stimuli (olfaction and gustation), potentially leading to loss of appetite at earlier stages, as well as impairment of autonomy following the progression of the disease or side effects of medication [91]. In general, a lower BMI before the onset of the disease cannot be considered a specific risk factor for ALS, although it seems that weight loss needs to be monitored and counteracted after ALS diagnosis, as it has consistently been associated with a worse prognosis [52,92,93].

## 4. Hypermetabolism in ALS

### Energy Expenditure

Energy homoeostasis represents the balance between energy intake from food and nutrients and energy expenditure, including resting energy expenditure (REE) and total daily energy expenditure (TDEE) [94]. An estimation of TDEE has not been standardized for ALS patients, although equations can now be used to estimate the energy requirements of ALS patients [95]. In a prospective multicenter study, REE was evaluated by indirect calorimetry in 80 ALS patients, and physical activity and body composition (lean and fat mass) were found to influence REE [95]. TDEE were estimated by Harris-Benedict, Mifflin-St Jeor, and Owen equations. Among these estimates, the Harris-Benedict equation was found to be the most practical formula for use in the care of ALS patients. The Harris-Benedict equation can estimate an individual’s daily calorie requirements, based on weight, height, age and sex, and taking activity level into account, to determine the TDEE [96]. However, this equation is most predictive when considering a normal body composition; therefore, it may not be accurate in ALS patients, who tend to be lean, with a normal or low BMI [97,98], and typically suffer from weight loss as the disease develops [99]. Thus, it is important to integrate the predictive validity of the equations to determine the energy needs with other methods, which may help to confirm the estimated requirements [100,101]. TDEE may be best estimated by using specific methods, such as indirect calorimetry and/or a double-labelled water system to assess REE and by taking correction factors into account (dietary-induced thermogenesis and physical activity) [102]. A comparison between the recorded values of REE with a theoretically calculated energy expenditure leads to a metabolism ratio, indicating hypermetabolism if ratio >1. Increased energy expenditure was reported in sporadic (*n* = 62) ALS [103,104], and 11 patients with familial ALS (fALS) [105]. In a study enrolling 26 Japanese ALS patients measuring the TDEE using the double-labelled water method for a 14-day period, a new logarithmic equation using the REE estimated by the Harris-Benedict equation and scores by the ALSFRS-R has been developed for ALS patients [106].

Hypermetabolism was found in ALS (up to 60% of patients), even with denervation and a rapid reduction in physical activity, which cause disuse muscle atrophy in ALS patients [103]. Thus, compensation for REE increase through diet is needed to avoid weight loss [107]. REE is positively correlated with muscle mass and inversely correlated with age, and is greater in men than in women [103]. TDEE seems to be significantly decreased with the progression of ALS stage and does not vary by sex [108]. A recent study, enrolling 315 ALS patients, showed that the trajectory of ALSFRS-R was not significatively influenced by a high or low REE [107]. In one study assessing REE in 58 ALS patients and 58 age- and sex-matched controls, ALS patients were more likely to have hypermetabolism than controls (OR = 5.4, 95% CI: 2.1 to 13.8, *p* < 0.01) [109]. During the 12 months of follow-up, there was a greater change in ALSFRS-R, which was inversely associated with hypermetabolic state (HR = 3.2, 95% CI: 1.1 to 9.4, *p* = 0.03) [109]. The hypermetabolism phenomenon was also studied in 62 patients and 31 healthy controls in a case-control study, in which REE in ALS patients was significantly higher than that of the control group (*p* = 0.03) [103]. The only laboratory variable that correlated with REE was the neutrophil count, although the reasons for this were unknown [103]. In another study, consecutive evaluations of REE from diagnosis to death in 44 ALS patients, REE levels remained higher than calculated values of an increase of 14%, followed by a trend for REE to decrease near death, whereas fat-free mass remained stable. Hypermetabolism was found in 62.3% of ALS patients. In multivariate analysis, REE was negatively linked to age and positively linked with fat-free mass [104]. A prospective 2-year longitudinal nutritional assessment in 61 ALS patients showed that about half of the patients were hypermetabolic, and hypermetabolism persisted even after adjustment for energy homeostasis over the follow-up period [110].

Hypermetabolism may lead to an anomalous lipid usage with a discrepancy between peripheric lipid stores’ mobilization and metabolic/nutritional status [103], although the underlying mechanisms are poorly understood. The possible reasons for such a conduction may include (i) the impact of the neurodegeneration of sympathetic nerve innervation of adipose tissue, which could affect the mobilization capacity of energy stores [64,111], which has been demonstrated, to some extent, to affect ALS patients [112]; (ii) higher energy expenditure for basic activities (i.e., breathing) due to muscle atrophy, fasciculations and spasticity [113]; (iii) mitochondria abnormalities with decreased energy production [114]; (iv) establishment of a pro-inflammatory state with increased production of pro-inflammatory cytokines by leukocytes [115,116]; (v) production of reactive oxygen species and increase in oxidative stress damage [117]. Among the aforementioned factors, mitochondrial metabolism was found to be hypermetabolic in ALS, involving both oxidative phosphorylation and glycolysis. This is not accompanied by an increase in mitochondrial ATP production, perhaps due to a compensatory adaptation to higher ATP demands [118]. It is intriguing to know that these cultured cells appear to show intrinsic mitochondrial or metabolic abnormalities in ALS. Although likely to involved in the hypermetabolism state, mitochondrial dysfunction in ALS is not ubiquitous in body cells [119]. Thus, we cannot exclude the possibility that it represents an epiphenomenon not causally related to ALS prognosis.

## 5. Malnutrition in ALS

Malnutrition is defined as weight loss exceeding 10% of the basal/previous weight or BMI < 18.5 [120]. Cachexia is a complex metabolic condition associated with an underlying disease, characterized by weight loss due to loss of muscle (with or without loss of FM), accompanied by a general inflammatory state [121]. Malnutrition can commonly be observed in ALS patients, with a prevalence rate of up to about 50% [113,122]. Using a BMI ≤ 18.5 as a cut-off, malnutrition was found in up to 20% of ALS patients, associated with a faster progression of motor symptoms, decreased survival time, and higher mortality risk [123,124]. In a French population-based study enrolling 322 ALS patients, those who had a weight loss of 10% or more at the time of diagnosis experienced a 45% increase in mortality risk compared to patients, experiencing a weight loss of lower than 5% or no weight loss [125].

A more recent prospective study from The Netherlands ALS Centre national database, investigating the prevalence and prognostic value of weight at the time of ALS diagnosis in 2420 patients, 67.5% of whom reported weight loss at first referral, showed that weight loss was a predictor of survival, with a 23% increased risk of death for every 10% increase in weight loss relative to body weight (HR 1.23, 95% CI: 1.13 to 1.51, *p <* 0.001) [93]. Similar results were shown in 63 ALS patients, for which the mean disease duration was significantly shorter in the subgroup of patients with weight loss exceeding 10% than in other patients [126]. On the other hand, stable weight at diagnosis was shown to be important, because patients who lost less than 5% of their usual body weight in the six months prior to ALS diagnosis had a higher median survival than those with higher body weight loss [61]. Out of the 40 ALS patients who were enrolled in a home network of surveillance, three patients were clinically malnourished and reported having unbalanced diets, with insufficient protein, high fat intake and not enough carbohydrate compared with French dietary recommendations [127]. In general, patients with ALS tend to assume an inadequate caloric intake, with about 15–16% fewer calories than recommended [99]. Poor dietary intake could be caused by dysphagia, taste disorders, or poor assistance being required during the meals [128]. Therefore, appropriate nutritional assessment and proper nutritional care is an important management aspect for patients with ALS [129].

## 6. Nutritional Epidemiology Studies of ALS

Table 2 shows epidemiological studies of macronutrients, micronutrients, and other food items in relation to the occurrence of ALS. Some studies showed that higher consumption of polyunsaturated fatty acids (PUFA) may play a protective role against ALS. A pooled analysis of five US cohorts (the Health Professionals’ Follow-up Study (HPFS), the Nurses’ Health Study (NHS), the Cancer Prevention II-Nutrition Cohort (CPS-II Nutrition), the Multiethnic Cohort Study (MEC) and the National Institutes of Health-AARP Diet and Health Study (NIH-AARP)) including more than 1 million individuals and a total of 995 ALS cases showed that a higher n-3 PUFA intake was associated with a 34% lower risk for ALS compared to the lowest intake (RR 0.66; 95% CI: 0.53 to 0.81; *p* for trend <0.001), with no evidence of association for n-6 PUFA [130]. Notably, both plant-derived alpha-linolenic acid (the main n-3 PUFA from vegetable sources) and marine n-3 PUFA were associated with lower ALS risk. The mechanisms underlying these findings include the ability of PUFA to modulate the cellular inflammatory response and act against oxidative stress [131]. Evidence of glutamate intake and risk of ALS is rather contrasting, with a study reporting higher risk [132] and another reporting null findings [133]. A population-based, case-control study in Netherlands showed that individuals with a higher preclinical intake of total fat (OR 1.14; 95% CI: 1.07 to 1.23; *p*  <  0.001), saturated fat (OR 1.43; 95% CI:1.25 to 1.64; *p*  <  0.001), trans-fatty acids (OR 1.03; 95% CI: 1.01 to 1.05; *p*  <  0.001), and cholesterol (OR 1.08; 95% CI: 1.05 to 1.12; *p*  <  0.001) were more likely to have ALS while a higher intake of alcohol was inversely associated with the likelihood of having ALS (OR 0.91; 95% CI: 0.84 to 0.99; *p* = 0.03) [134]. Another study conducted in three Italian regions involving 212 cases and matched controls showed an inverse association between coffee and tea (OR = 0.29, 95% CI 0.14 to 0.60), whole bread (OR  =  0.55, 95% CI 0.31 to 0.99), raw vegetables (OR  =  0.25, 95% CI 0.13 to 0.52) and citrus fruits (OR  =  0.49, 95% CI 0.25 to 0.97) and likelihood of having ALS, and a direct association with red and processed meat (OR  =  2.96, 95% CI 1.46 to 5.99 and OR  =  3.87, 95% CI 1.86 to 8.07, respectively), increased total and animal protein intake (OR  =  2.96, 95% CI 1.08 to 8.10 and OR  =  2.91, 95% CI 1.33 to 6.38, respectively), sodium (OR  =  3.96, 95% CI 1.45 to 10.84), zinc (OR  =  2.78, 95% CI 1.01 to 7.83) and glutamic acid (OR  =  3.63, 95% CI 1.08 to 12.2) [135]. Another case-control study found a negative association with high carbohydrate intake [136], but this was not confirmed in two population-based studies [132,133]. There are inconclusive results regarding the relationship between vitamin E consumption and risk of ALS: an inverse association was reported in a case-control [137] and a cohort study [138] but null results were found in two other case-control studies [139,140]. A recent pooled analysis showed lower ALS rates among individuals with the highest dietary vitamin E intake, but in multivariable adjustment, this finding was no longer significant [141]. However, increasing years of supplementation with vitamin E was inversely related with the likelihood of having ALS [141].

The intake of carotenoids and ALS risk has been investigated in case-control studies [139,140] reporting a lower risk of ALS among individuals with higher lycopene intake [139] and inverse ALS rates across quartiles of beta-carotene [140]. Further evidence has been ported in a pooled analysis of five prospective cohorts, accounting for a total of 1153 ALS deaths among more than 1,000,000 participants, showing an inverse relationship between total intake of carotenoids, beta carotene and lutein and ALS risk [142]. Other investigated micronutrients, such as calcium, magnesium and vitamin C, have shown no association with risk of ALS [139,142,143]. A recent cross-sectional study, including 302 newly diagnosed ALS patients, reported that antioxidants, carotenes, and their major food sources, fruits and vegetables, were associated with higher ALS function; specifically, lutein, zeaxanthin, and n-3 PUFA intake was associated with ALSFRS-R score, and n-3 and n-6 PUFA intake was associated with FVC percentage [144]. Regarding animal-derived foods, a study reported that survival time was associated with fat, protein, and meat intake [145].

The putative protective action observed for n-3 PUFA, carotenoids, and Vitamin E could be due to the antioxidant activity of these micronutrients [146], as oxidative stress is a well-described phenomenon in ALS [147,148]. These epidemiological studies suggest that antioxidative micronutrients are associated with lower risk of ALS and better motor function in patients with ALS. It may be worth accounting for the individual genetic risk factors which modulate the metabolism of any macro- or micronutrient; for instance, a genetic variation in the beta-carotene monooxygenase gene could influence the bioavailability of this micronutrient [149].

## 7. Conclusions

Metabolic and nutritional factors seem to play a role in some of the mechanisms involved in ALS development. Notably, energy metabolism appears impaired and energy expenditure is increased to a hypermetabolic state in a higher proportion of ALS patients. However, the causes of these abnormalities are still unclear, and only a few studies have investigated the energy metabolism of patients before the development of ALS. Metabolic changes in patients with ALS occur from the onset and throughout disease progression, and whether the changes precede the diagnosis is unknown. Overall, the observed hypermetabolic state and weight loss may lead to muscle loss and cachexia. Thus, weight maintenance in normal weighted patients or weight gain in underweighted ALS patients should lead to better prognosis. Importantly, an estimation of the energy requirements should take the hypermetabolic state and possible increased needs into account. There is not enough evidence to support any specific dietary intervention regarding macronutrient or food content, or antioxidant supplement formulation over another. However, since ALS may lead to cachexia at the end stages, the preparation of nutritionally adequate meal plans with the inclusion of antioxidant and anti-inflammatory (and limited pro-inflammatory) dietary factors may be advisable. Thus, nutritional evaluation of ALS patients should be included at the first referral of the ALS patient and continued during the follow-up visits in order to ensure adequate energy and nutrient intake over the time of disease progression. Further studies are needed to better investigate the metabolic and nutritional aspects related to the mechanisms of ALS and to provide better nutritional care in patients with ALS. Future studies, including genetic, biochemical, and nutritional investigations, are of paramount importance to clarify the role of nutrition in ALS.

## Figures and Tables

**Table 1:** Case series and case-control studies investigating lipid and glucose metabolism in ALS.

Authors, and Date of Publication	Study Design	Number of ALS/Controls	Most Important Lipids and Glucose Laboratory Values Considered	Main Findings
Gustafson, 1972 [47]	Case-control	63/76HC	Serum cholesterol, triglycerides, serum lipoprotein patterns	Hypercholesterolemia and hypertriglyceridemia in 27% and 10% of ALS, respectively.
Harno, 1984 [29]	Case-control	21/10 OND	OGTT, insulin levels, insulin resistance	Higher basal insulin levels in ALS than in controls (*p* < 0.05), but no difference after OGTT.
Reyes 1984 [27]	Case-control	10/15 HC/4 OND	OGTT, insulin levels and resistance	No difference in terms of IGT. Insulin resistance in ALS vs. two groups (*p* < 0.001).
Perurena, 1987 [28]	Case-control	10/16 HC	Insulin levels	Plasma insulin levels were not elevated in ALS.
Dupuis, 2008 [48]	Case-control	369/286HC	Serum cholesterol, triglycerides, HDL, LDL, and LDL/HDL ratio	Higher cholesterol, triglycerides, LDL and LDL/HDL ratio in ALS patients than controls (*p* < 0.001). Longer survival (by ~12 months) in ALS with high LDL/HDL ratio vs. low LDL/HDL (*p* < 0.05).
Chio’, 2009 [49]	Case-control	658/658 HC	Serum cholesterol, triglycerides, HDL, LDL, and LDL/HDL ratio	No differences ALS vs. controls. Total cholesterol, LDL/HDL ratio, and triglycerides levels showed a significant decrease in patients with FVC < 70% vs. FVC ≥ 90% (*p* < 0.05).
Pradat, 2010 [46]	Case-control	21/21 HC	OGTT, insulin levels, serum cholesterol, triglycerides, HDL, LDL, FFAs, TNF-α and IL-6, leptin and adiponectin	At 120 min blood glucose was higher in ALS vs. controls (*p* = 0.006). Insulin levels were increased in ALS patients with IGT vs. no IGT patients at 60 and 90 min after glucose load (*p* = 0.045 and *p* = 0.044, respectively). FFA levels were higher in patients with IGT vs. no IGT (*p* = 0.04). IGT was not associated with disease duration or severity.
Dorst, [50]	Retrospective database study	488/-	Serum cholesterol, triglycerides, LDL, HDL, LDL/HDL ratio	Hypertriglyceridemia was associated with longer survival (14 months *p* = 0.006). Hypercholesterolemia was associated with longer survival (11 months, *p* = 0.047).
Sutedja, 2011 [51]	Case-control	303/2100 HC	Serum cholesterol, triglycerides, LDL, HDL, LDL/HDL ratio	In ALS less use of cholesterol-lowering drugs (*p* = 0.008), lower BMI (*p* = 0.001), lower LDL/HDL ratio (women: *p* < 0.001; men: *p* < 0.001) in ALS. Cholesterol and LDL were lower in patients with FVC < 70% (*p* < 0.05). No correlation hyperlipidemia with survival.
Paganoni, 2011 [52]	Retrospective database study	427/-	Serum cholesterol, triglycerides, LDL, HDL, LDL/HDL ratio	Hyperlipidemia was not associated with increased survival.
Ikeda, 2012 [53]	Case-control	92/92	Serum cholesterol, triglycerides, LDL, HDL, LDL/HDL ratio, fasting blood sugar	Women with ALS had increased levels of cholesterol (*p* < 0.05), LDL (*p* < 0.01), and triglycerides (*p* < 0.01). The annual decline in ALS-FRS and FVC was inversely correlated with baseline levels of cholesterol (*p* < 0.001) and LDL (*p* < 0.001).
Dedic, 2012 [54]	Retrospective database study	82/-	Serum cholesterol, triglycerides, LDL, HDL, LDL/HDL ratio	Hyperlipidemia was not associated with increased survival.
Yang, 2013 [55]	Case-control	95/99	Serum cholesterol, triglycerides, LDL, HDL, LDL/HDL ratio, glucose	Serum cholesterol (*p* = 0.004), LDL (*p* = 0.040), triglycerides (*p* = 0.025), LDL/HDL ratios (*p < 0*.001; only in men) were significantly lower than controls.
Huang, 2015 [56]	Case-control	413/400	Serum cholesterol, LDL, HDL, triglycerides	Longer life expectancy (median 5.8 months) in ALS patients with higher triglyceride levels (>127.5 mg/dl).
Mandrioli, 2017 [57]	Retrospective cohort study	275/-	Serum cholesterol, triglycerides, LDL, HDL, LDL/HDL ratio, glucose, ferritin, albumin, creatinine	Triglycerides were inversely associated with the odds of death or tracheostomy (*p* < 0.05).
Wei, 2017 [38]	Prospective cohort study	450/-	fasting HbA1c and glucose	ALS with higher baseline HbA1c had significantly higher risks of mortality (*p* < 0.001).
Mariosa, 2017 [32]	Population cohort study	623/15,575	Serum glucose, serum cholesterol, triglycerides, apoB and apoA-I, LDL and HDL	Increase in LDL (*p* < 0.001), apoB (*p < 0*.001), and apoB/apoA-I ratio (*p* < 0.001) was associated with a higher incidence of ALS. High glucose level (≥ 6.11mmol/L) was associated with a lower incidence (*p* < 0.001).
Delaye, 2017 [58]	Case-control	30/30	Serum cholesterol, 11 distinct lipoprotein subclasses	Higher total cholesterol, HDL, and LDL levels in ALS patients than controls (*p* < 0.001, *p* < 0.001, and *p* = 0.0065, respectively).
Ingre, 2020 [59]	Cohort study	99	Cholesterol, lipoproteins	Increase in total cholesterol (*p* = 0.01), LDL (*p* = 0.02), LDL:HDL ratio (*p* = 0.02), apolipoprotein B (*p* = 0.01), or apolipoprotein B/apolipoprotein AI ratio (*p* < 0.01) was associated with a lower risk of death in ASL patients.

ALS, amyotrophic lateral sclerosis; Apo, Apolipoprotein; FFAs, free fatty acids; FVC, forced vital capacity; HbA1c, glycated haemoglobin; HC, healthy controls; HDL, high-density lipoprotein; IGT, impaired glucose tolerance; IL-6, interleukin-6; LDL, low-density lipoprotein; OND, other neurological diseases; OGTT, oral glucose tolerance test; TNF-α, tumor necrosis factor-alpha.

**Table 2:** Summary of studies on food intake in ALS.

Authors, and Date of Publication	Study Design, Country	Number of ALS/Controls	Macro and Micronutrients Investigated	Main Findings
Nelson, 2000 [132]	Case-control, US	161/321	carbohydrates, proteins, fats, vitamins and minerals	High intake of glutamate (>15.07 g/day), saturated fats (*p <* 0.001), and PUFA (*p <* 0.001) were associated with ALS.
Longnecker, 2000 [139]	Case-control, US	107/262	calcium, magnesium, vitamins	No positive or negative association with ALS.
Ascherio, 2005 [138]	Mortality surveillance in a Cancer cohort, US	525/957,740	vitamins E and C	Age- and smoking-adjusted relative risk to develop ALS in regular users of vitamin E for 10 or more years was higher than non-users (*p <* 0.001).
Okamoto, 2007 [136]	Case-control, Japan	153/306	carbohydrates, saturated and unsaturated fat	Carbohydrate intake (>295 g per day) was directly associated with ALS (*p <* 0.001); PUFA (*p <* 0.001), saturated fatty acids (*p <* 0.001) and monounsaturated fatty acids (*p <* 0.001) were inversely associated with ALS.
Veldink, 2007 [137]	Case-control, Netherlands	132/220	PUFAs, glutamate, flavonol, lycopene, vitamin C, vitamin B2, vitamin E calcium or phytoestrogens	PUFAs and vitamin E intake were inversely associated with ALS (*p <* 0.001 and *p <* 0.001, respectively).
Morozova, 2008 [133]	Mortality surveillance Cancer cohort, US	841/over 1 million	carbohydrates, proteins, fats, vitamins and minerals	Consumption of chicken was inversely related to risk of ALS (*p <* 0.001).
Okamoto, 2009 [140]	Case-control, Japan	153/306	fruits and vegetables	Not significant lower odds of ALS with intake of fruit and vegetables.
Wang, 2011 [141]	Pooled analysis of data from 5 prospective cohorts, US	805/1,055,546	vitamin E	ALS rates declined with longer years of use of vitamin E (*p* for trend = 0.01).
Fitzgerald, 2013 [142]	Pooled analysis of data from 5 prospective cohorts, US	1153/1,100,910	vitamin C and carotenoids	Total carotenoids and lutein intake was associated with decreased risk of ALS (*p* for trend = 0.004 and *p* for trend= 0.01, respectively). Lycopene, β-cryptoxanthin, and vitamin C were not associated with decreased risk of ALS.
Fitzgerald, 2014 [130]	Pooled analysis of data from 5 prospective cohorts, US	995/1,002,082	dietary fat intakes	N-3 PUFA intake was associated with a lower risk for ALS (*p* for trend < 0.001). The observed reduced risk was found for both ALA (*p* for trend = 0.003) and marine n-3 PUFA intake (*p* for trend = 0.03).
Fondell, 2013 [143]	Pooled analysis of data from 5 prospective cohorts, U.S.	1093/1,050,000	dietary magnesium intake	Dietary intake of magnesium was not associated with ALS risk.
Park, 2015 [106]	Case study, Korea	193/-	carbohydrates, proteins, fats, vitamins and minerals	Patients in the lowest values of ALSFRS-R consumed fewer vegetables, grains, seasonings, oils, and meats than others, but higher amounts of fruits and beans.
Nieves, 2016 [144]	Cross-sectional baseline analysis on ALS cohort, US	302/-	carbohydrates, proteins, fats, vitamins and minerals	Increased consumption of antioxidants and carotenoids was associated with higher ALSFRS-R scores or percentage FVC.
Pupillo, 2018 [135]	Case-control, Italy	212/212	carbohydrates, proteins, fats, vitamins and minerals	Higher intake of coffee, tea, whole bread, raw vegetables, and citrus fruits was associated with significantly higher ALSFRS-R scores or percentage FVC. Higher risk was associated with higher consumption of red and processed meat, total and animal protein, sodium, zinc, and glutamic acid.
Kim, 2020 [145]	Retrospective	148/-	carbohydrates, proteins, fats	Fat, protein, and meat intake was positively associated with survival time of ALS patients.
